# Correlation analysis of the impact of *Clonorchis sinensis* juvenile on gut microbiota and transcriptome in mice

**DOI:** 10.1128/spectrum.01550-24

**Published:** 2024-12-27

**Authors:** Xueling Deng, Shitao Li, Yuhong Wu, Jiali Yao, Wei Hou, Jiangyao Zheng, Boying Liang, Xiaole Liang, Qiping Hu, Zhanshuai Wu, Zeli Tang

**Affiliations:** 1 Department of Cell Biology and Genetics, School of Basic Medical Sciences, Guangxi Medical University, Nanning, China; 2 Key Laboratory of Longevity and Aging-related Diseases of Chinese Ministry of Education, Guangxi Medical University, Nanning, China; 3 Key Laboratory of Basic Research on Regional Diseases (Guangxi Medical University), Education Department of Guangxi Zhuang Autonomous Region, Nanning, China; 4 Guangxi Key Laboratory of Thalassemia Research, Nanning, China; 5 Department of Immunology, Guangxi University of Chinese Medicine, Nanning, China; 6 Guangxi Key Laboratory of Translational Medicine for treating High-Incidence Infectious Diseases with Integrative Medicine, Nanning, China; Yangzhou University, Yangzhou, Jiangsu, China

**Keywords:** *Clonorchis sinensis*, juvenile, mouse, early infection, gut microbiota, transcriptome

## Abstract

*Clonorchiasis* remains a non-negligible global zoonosis, imposing serious socio-economic burdens in endemic regions. The interplay between gut microbiota and the host transcriptome is crucial for maintaining health; however, the impact of *Clonorchiasis sinensis* juvenile infection on these factors is still poorly understood. This study aimed to investigate their relationship and potential pathogenic mechanisms. The BALB/c mouse model of early infection with *C. sinensis* juvenile was constructed. Pathological analyses revealed that *C. sinensis* juvenile triggered liver inflammation, promoted intestinal villi growth, and augmented goblet cell numbers in the ileum. Additionally, the infection altered the diversity and structure of gut microbiota, particularly affecting beneficial bacteria that produce short-chain fatty acids, such as *Lactobacillus* and *Muribaculaceae*, and disrupted the Firmicutes/Bacteroidetes ratio. Gut transcriptome analysis demonstrated an increase in the number of differentially expressed genes (DEGs) as infection progressed. Enriched Gene Ontology items highlighted immune and detoxification-related processes, including immunoglobulin production and xenobiotic metabolic processes. Kyoto Encyclopedia of Genes and Genomes pathway analysis further indicated involvement in circadian rhythm, as well as various detoxification and metabolic-related pathways (e.g., glutathione metabolism and glycolysis/gluconeogenesis). Prominent DEGs associated with these pathways included Igkv12-41, Mcpt2, Arntl, Npas2, Cry1, and Gsta1. Correlation analysis additionally identified *Bacteroides_sartorii* as a potential key regulator in the interaction between gut microbiota and transcriptome. This study sheds light on the alterations in gut microbiota and transcriptome in mice following *C. sinensis* juvenile infection, as well as their correlation, laying a foundation for a better understanding of their interaction during infection.

**IMPORTANCE:**

This study highlighted the impact of *C. sinensis* juvenile infection on the gut microbiota and transcriptome of BALB/c mice. It induced liver inflammation, promoted intestinal villi growth, and altered goblet cell numbers. The infection also disrupted the diversity and structure of gut microbiota, particularly affecting beneficial bacteria. Transcriptome analysis revealed increased expression of genes related to immune response and detoxification processes. Important pathways affected included circadian rhythm, glutathione metabolism, and glycolysis/gluconeogenesis. Notable genes implicated included Igkv12-41, Mcpt2, Arntl, Npas2, Cry1, and Gsta1. *Bacteroides_sartorii* emerged as a potential key regulator in this interaction.

## INTRODUCTION

*Clonorchis sinensis* is a liver fluke that can cause human clonorchiasis, which can lead to various health problems including chronic inflammation, bile duct epithelial hyperplasia, periductal fibrosis, and even cholangiocarcinoma (1, 2). The World Health Organization has classified *C. sinensis* as a Group I carcinogen (3). Human infection with *C. sinensis* is mainly caused by the consumption of raw or undercooked freshwater fish that contain metacercariae (1, 2). Once ingested, the metacercariae excyst in the duodenum and migrate to the bile ducts (4, 5). The juveniles then develop into adults in the human bile duct over a period of about 1 month (4, 5). Throughout the process of parasitism and development, *C. sinensis* releases various proteins, including excretory-secretory products (*Cs*ESPs), which can penetrate the ulcerative bile duct wall into the surrounding liver tissue and induce liver injury. Additionally, these parasite-derived proteins can also affect the composition of the gut microbiota through bile excretion along the bile duct to the gut lumen (5, 6).

The gut microbiota plays a crucial role in maintaining gut health by acting as a barrier against harmful microorganisms (7). It produces various metabolic products, including short-chain fatty acids (SCFAs), that help maintain gut homeostasis and overall health (8, 9). Previous studies have reported that parasitic infections, such as *Schistosoma japonicum*, *Toxoplasma gondii*, and *C. sinensis* infection, significantly impact the abundance of the host gut microbiota (10–12). Increasing evidence shows a close relationship between the gut microbiota and the transcriptome. For example, dysregulation of host gene expression and pathways in colorectal cancer have been shown to contribute to the development of the disease (13). The gut microbiota has been identified as a potential regulator of host gene expression in model organisms, and it can also influence the epigenetic programming host gut gene, particularly in immune and metabolic processes (14, 15). Furthermore, *in vitro* cell culture experiments have demonstrated that specific gut microbiota can alter gene expression in human colonic epithelial cells (16).

Gut barrier disorders and dysbiosis contribute to the development of various liver diseases, including fibrosis, cirrhosis, and cancerous transformation (17). Therefore, it is essential to comprehend the interactions between the host gut microbiome and gene regulation in order to understand the pathogenesis of hepato-gut axis-related diseases (18, 19). The objective of this study was to investigate the impact of *C. sinensis* juvenile infection on the tissue structure, microbiota, and transcriptome of mice intestines. Additionally, we aimed to explore the relationship between the microbiota and transcriptome to gain insights into potential pathogenic mechanisms. The findings of this study will contribute to the understanding of *C. sinensis* juvenile infection and its impact on the gut microbiota, while also laying the groundwork for studying changes in the gut microbiota caused by other parasitic infections.

## MATERIALS AND METHODS

### Parasites

*C. sinensis* metacercariae were obtained from naturally infected freshwater fish (*Pseudorasbora parva*) in Heng County, Guangxi Zhuang Autonomous Region, China. Surimi, after being prepared by removing fish bones, fins, tails, scales, and viscera, was then placed in a 0.8% pepsin solution containing 0.2% HCl, and it was left to digest overnight at 37°C. Afterward, the mixture was filtered through a 60–80 mesh sieve. Finally, the living metacercariae were isolated from clean sediment using light microscopy and stored in PBS at 4°C (20).

### Animals and experimental design

Twenty specific pathogen-free female BALB/c mice (6 weeks old) were purchased from Hunan SJA Experimental Animal Co., Ltd. Before experiment, the mice were adapted to the experimental environment for 1 week. All animals were well maintained in a temperature-controlled room (25°C ± 2°C) with a 12:12 hour dark/light cycle and fed standard chow (standard chow main ingredients: corn, soybean meal, flour, bran, calcium hydrogen phosphate, fish meal, stone powder, sodium chloride, vitamin premix, trace element premix, etc.). The mice were randomly divided into control (0 d) group, 1-day (1 d) group, 3-day (3 d) group, and 7-day (7 d) group (*n* = 5 per group). All mice, except for control mice, were given 60 metacercariae via gavage at the same time point. The control group mice were just orally administered with the same volume of PBS (200 µL). The mice were then fed according to the experimental schedule for 0, 1, 3, and 7 days, respectively.

Mice were sacrificed at 0 d, 1 d, 3 d, and 7 d post-gavage infection, and corresponding specimens were collected. First, fresh feces from mice in each group were collected and immediately snap-frozen and stored in liquid nitrogen for subsequent determination of gut microbiome (*n* = 5 per group). Then, the ileum tissues of mice in each group were quickly frozen in liquid nitrogen for RNA sequencing (with random sampling of *n* = 3 per group), and a segment of ileum and liver tissues was fixed in 4% paraformaldehyde for histopathological staining. The time range of this study is from June 2022 to February 2024.

### Histology staining

Liver and ileum tissues from mice in each group were embedded in paraffin after fixation with 4% paraformaldehyde and dehydration. The tissues were then cut into 5 µm sections, and H&E and AB-PAS trichrome staining were performed respectively. Subsequently, the stained sections were observed and photographed under an optical microscope.

### Gut microbiota analyses

Feces from the 0 d, 1 d, 3 d, and 7 d groups (*n* = 5 per group) were subjected to 16s rRNA sequencing. For 16s rRNA sequencing, genomic DNA was first extracted from feces according to the instructions of EZNA Soil DNA Kit (Omega Bio-Tek, Norcross, GA, USA), then DNA purity was determined by NanoDrop2000 (Thermo Fisher Scientific, Waltham, USA) and agarose and integrity were subjected to gel electrophoresis. Subsequently, Quantifluor (Promega, Lyon, France) was used to perform PCR amplification of the V3-V4 region of 16S rRNA (338F: 5′-ACTCCTACGGGAGGCAGCAG-3′, 806R: 5′-GGACTACHVGGGTWTCTAAT-3′), and the amplified products were detected by 2% gel electrophoresis and recovered by AxyPrep DNA Gel Extraction Kit (Axygen, USA). Sequencing was performed using Illumina’s Miseq PE300/NovaSeq PE250 platform (Shanghai Meiji Biomedical Technology Co., Ltd., Shanghai, China). Amplicon sequence variants were sequences obtained after denoising by DADA2 (or Deblur). Finally, the data were analyzed using the online cloud platform (https://cloud.majorbio.com/) of Majorbio (Shanghai, China). Analysis content included alpha diversity (Ace, Chao, Shannon, and Simpson) and principal coordinates analysis (PCoA), Venn diagram analysis, community composition analysis, differential species analysis, and Linear discriminant analysis Effect Size (LEfSe) (http://huttenhower.sph.harvard.edu/LEfSe).

### Transcriptomic analyses

To evaluate the gene expression profile of mouse gut, total RNA of ileum tissues was extracted from the 0 d, 3 d, and 7 d groups (with random sampling of *n* = 3 per group) using MJzol reagent (Invitrogen, MA, USA), respectively. The Illumina Novaseq 6000 platform (San Diego, CA, USA) was used for sequencing of the samples. After quality control, clean data (reads) were obtained for alignment to the reference genome. Differentially expressed genes (DEGs) were obtained using the Benjamini and Hochberg (BH) method of DESeq2 screening, and hierarchical clustering was performed using the Euclidean distance and average linkage method. Finally, Goatools and KOBAS software were used to perform enrichment analysis, including Gene Ontology (GO, http://geneontology.org/) term analysis and Kyoto Encyclopedia of Genes and Genomes (KEGG, http://www.genome.jp/kegg//) pathway enrichment analysis.

### Correlation analyses between gut microbiome and transcriptome

In order to better understand the relationship between gut microbiota and gut genes, Spearman correlation analysis was used to reveal the correlation between the gut microbiome and DEGs enriched to significant KEGG pathways. At the same time, Spearman correlation analysis was used to display the relationship between microbiota and DEGs of specific pathway based on the absolute value of the correlation coefficient being greater than 0.6 and *P* < 0.05.

### Statistical analyses

For microbiota data, Wilcoxon rank-sum test and false discovery rate (FDR) test correction were calculated to test alpha diversity indicators, PCoA used the Bray-Curtis distance algorithm to calculate the distance between samples, and ANOSIM was used to test differences between groups. Wilcoxon rank-sum test and FDR test correction between two groups were used to detect differences between groups, and LEfSe analysis (LDA > 2, *P* < 0.05) was used to identify microbiota taxa with significant differences in abundance from genus to species levels between different groups. The above data were all calculated using R-3.3.1. For transcriptomic data, DESeq2 software was used for DEGs analysis, and the BH method was used for multiple testing correction of *P* values. The filtering criteria for DEGs were set to *P*adjust <0.05 and |logFC| ≥ 2.0. Fisher’s exact test was used for both GO and KEGG analyses. The significance of genes enrichment in GO analysis was determined based on a *P*adjust <0.05, while *P* value or *P*adjust <0.05 was used for KEGG analysis. Correlation network diagram analysis was conducted between gut microbiotas and DEGs based on Spearman correlation (|r| > 0.6, *P* < 0.05).

## RESULTS

### Histopathological changes in mouse liver and gut tissues caused by *C. sinensis* infection

**FIG 1:**
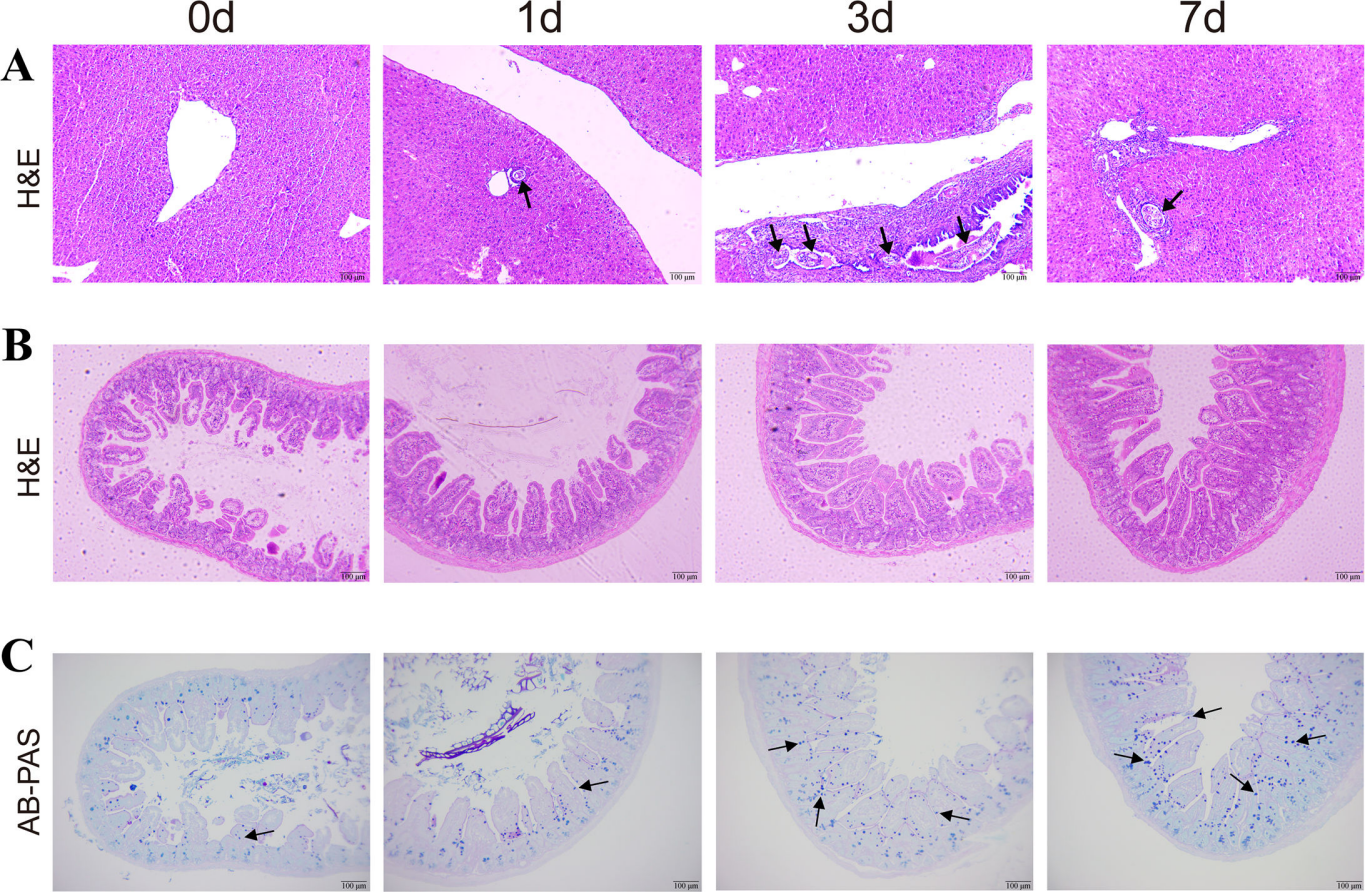
Histopathological changes in liver and gut tissues infected with *C. sinensis* at different time points (× 100). (**A**) Liver sections of *C. sinensis*-infected mice were stained with H&E staining. Thick black arrows represent juvenile worms. Ileum tissue sections of *C. sinensis*-infected mice were stained with H&E (**B**) and AB-PAS staining (**C**), respectively. Black arrows represent goblet cells.

The results of H&E staining revealed the worsening of inflammatory cell infiltration with prolonged infection time, with bold black arrows indicating juvenile parasitism in the liver (Fig. 1A). H&E staining of ileum tissues results showed that no obvious pathological changes were observed in *C. sinensi*s juvenile within 7 days of infection; however, as the infection time progressed, the ileal villi elongated (Fig. 1B). Meanwhile, AB-PAS results demonstrated an increase in the number of goblet cells and secretion of acidic mucus (indicated by black arrows) in the ileum as the infection time increased (Fig. 1C).

### Effects of *C. sinensis* on the diversity of gut microbiota in mice

**FIG 2:**
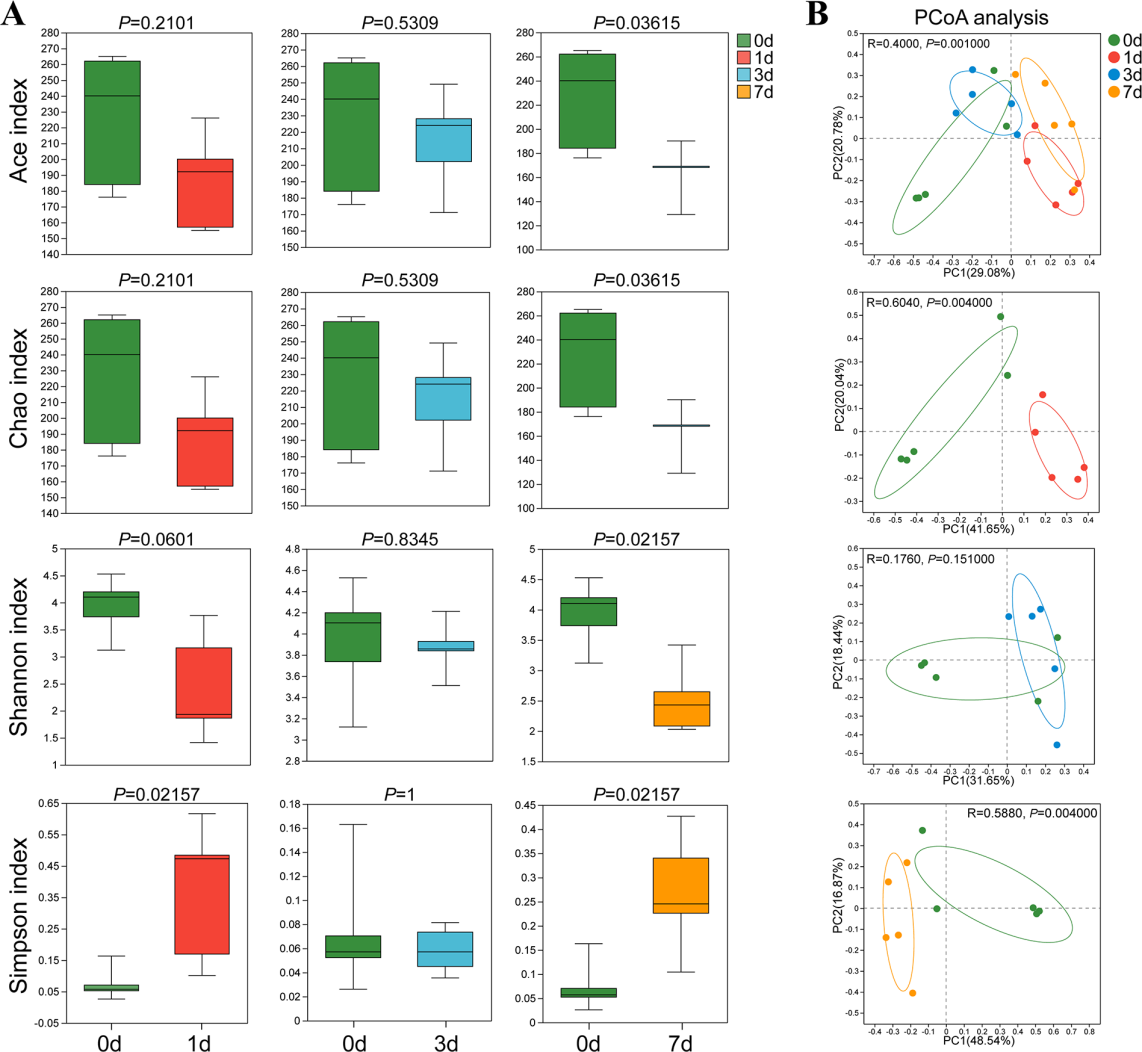
Changes in the diversity of gut microbiota in mice induced by *C. sinensis* at different time points. (**A**) The comparison of Ace, Chao, Shannon, and Simpson index between 1 d vs 0 d, 3 d vs 0 d, and 7 d vs 0 d. (**B**) PCoA of 1 d vs 0 d, 3 d vs 0 d, and 7 d vs 0 d.

The rank abundance curve showed that compared with control group, the distribution of 1 d, 3 d, and 7 d groups was less uniform, and the microbial richness was also reduced (Fig. S1). The results of alpha diversity showed that compared to the control group, as the infection time increased, the Ace, Chao, and Shannon indices generally exhibited a decreasing trend, while the Simpson index showed the opposite trend, especially the 7 d groups (Ace, *P* = 0.03615, Chao, *P* = 0.03615, Shannon, *P* = 0.02157, Simpson, *P* = 0.02157) (Fig. 2A). PCoA showed that samples were significantly separated among four groups (*P* = 0.001). Among them, the differences between the 1 d vs 0 d and 7 d vs 0 d were significant (both *P* = 0.004) (Fig. 2B).

### Changes in the gut microbial community structure of mice infected with *C. sinensis*

**FIG 3:**
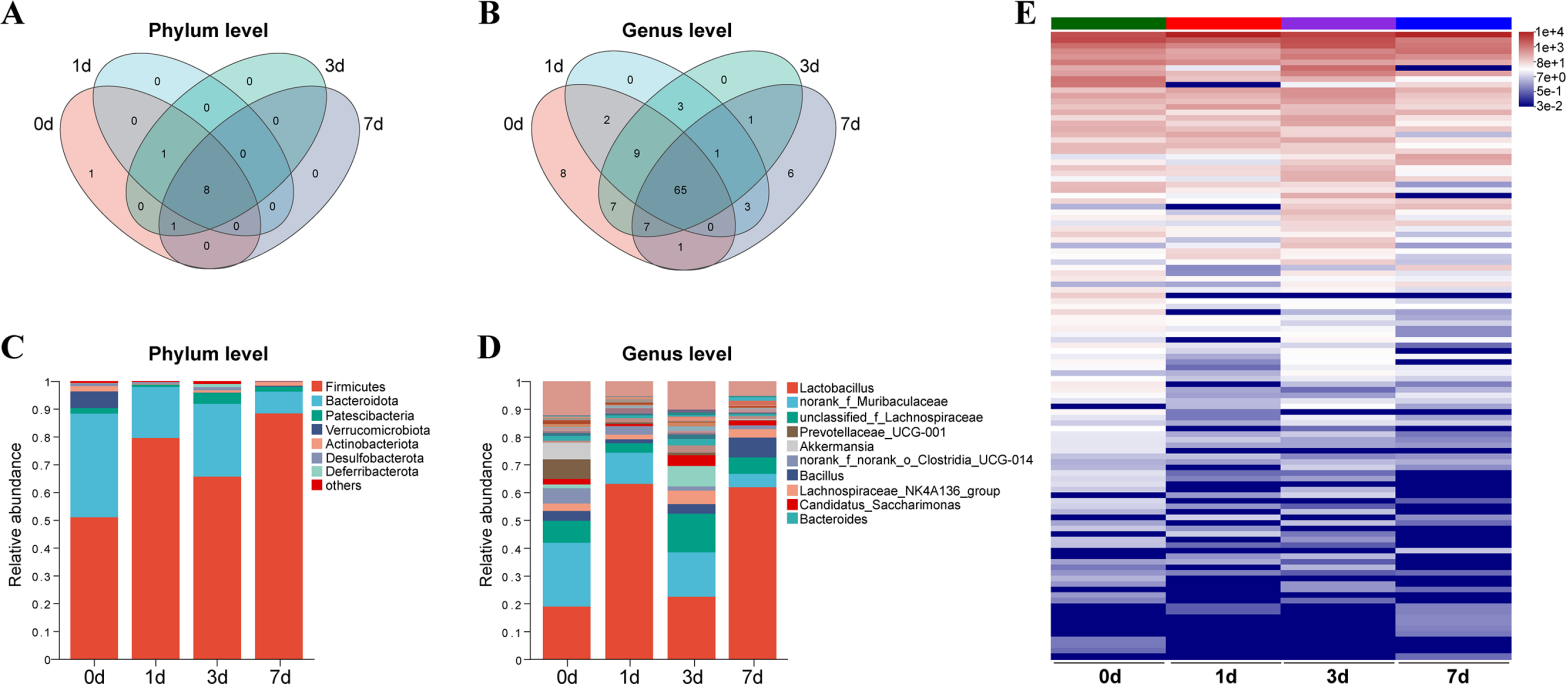
Changes in the levels of gut microbiota induced by *C. sinensis* at different time points of infection. (**A**) Venn analysis of gut microbiota at the phylum level. (**B**) Venn analysis of gut microbiota at the genus level. (**C**) Relative abundance of gut microbiota at the phylum level. (**D**) Relative abundance of gut microbiota at the genus level. (**E**) Genus level expression of gut microbiota at different time points of infection.

To further understand the impact of *C. sinensis* juvenile infection on the composition and structure of gut microbiota. The results of the Venn analysis displayed the microbial number of gut microbiota at the phylum and genus levels (Fig. 3A and B). At the phylum level, the four groups of microbial combinations were mainly Firmicutes, Bacteroidetes, Proteobacteria, and Verrucomicrobiota. Compared with control group, the abundance of Firmicutes increased, while Bacteroidetes decreased. The *C. sinensis* juvenile infection had the greatest impact on Verrucomicrobiota abundance. In addition, compared with the control group, the ratio of Firmicutes/Bacteroidetes (F/B) in the infected groups increased (Fig. 3C). At the genus level, following juvenile infection of *C. sinensis*, there was a shift in the dominant bacterial genus from *norank_f_Muribaculaceae* of Bacteroidetes to *Lactobacillus* of Firmicutes. And the abundance of *Prevotellaceae_UCG-001*, *Akkermansia*, and *norank_f_norank_o_Clostridia_UCG-014* varied greatly (Fig. 3D). The heatmap showed the overall expression of all detected bacterial genera in each group (Fig. 3E).

### Differences in microbial species of gut microbiota in mice infected with *C. sinensis*

**FIG 4:**
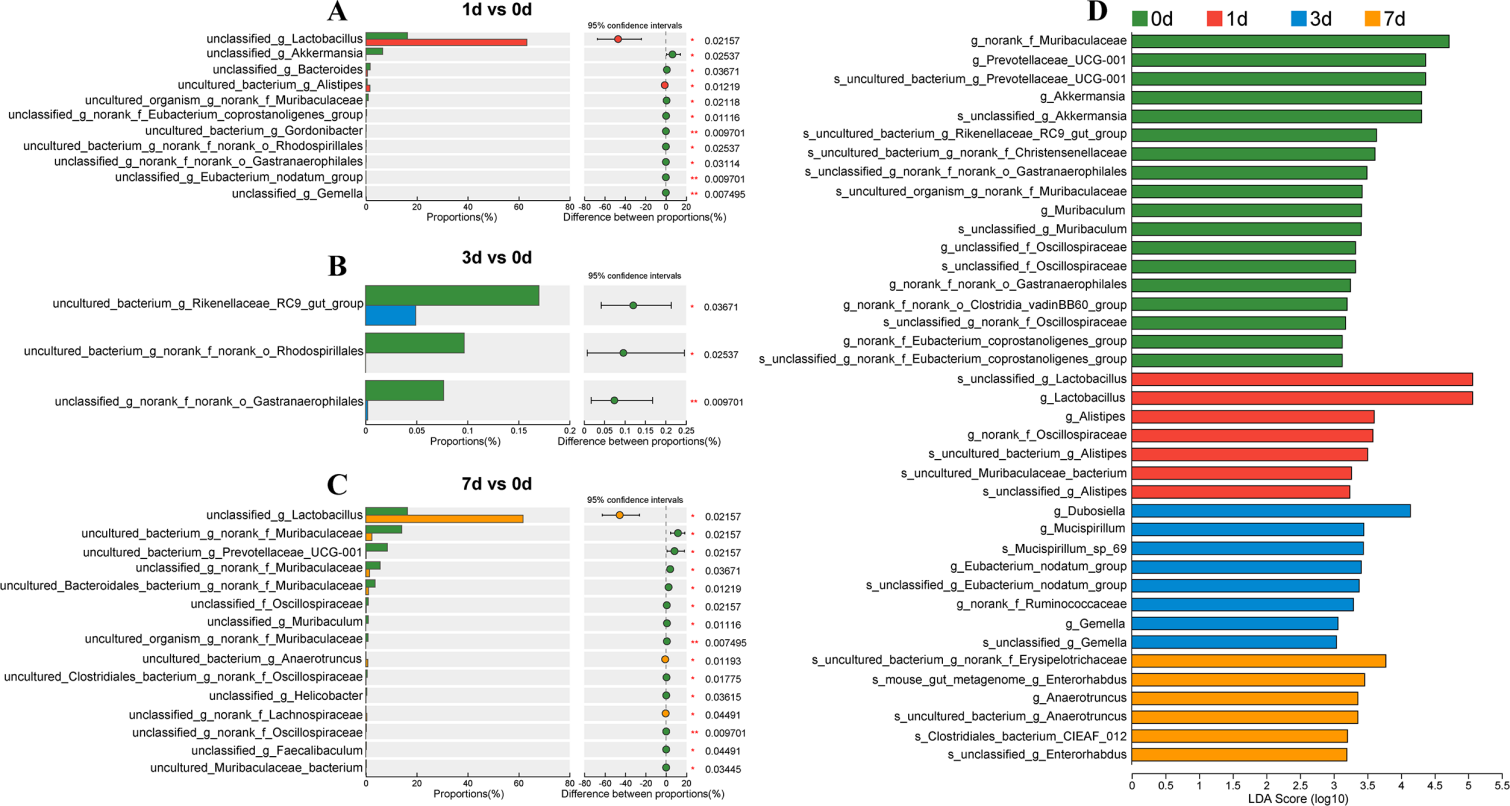
The changes in gut microbiota at different time points of infection with *C. sinensis*. Differences in gut microbiota between 1 d vs 0 d (**A**), 3 d vs 0 d (**B**), and 7 d vs 0 d (**C**). (**D**) LEfSe bar plot of gut microbiota at various time points after infection.

To assess the significance level of species richness differences, abundance analysis of microbiota in each group was conducted. Compared with the control group, on 1 d post infection, a greater impact on potential beneficial bacteria, such as *uncultured_bacterium_g_Gordonibacter*, *unclassified_g_Lactobacillus*, *uncultured_organism_g_norank_f_Muribaculaceae*, etc., was observed. (Fig. 4A). On 3 d post infection, the differences between bacterial species decreased (Fig. 4B). On 7 d post infection, the number of different bacterial species increased, especially in the abundance of beneficial bacteria such as *uncultured_bacterium_g_Anaerotruncus*, and *unclassified_g_Lactobacillus,* significantly. The abundance of *uncultured_organism_g_norank_f_Muribaculaceae*, *unclassified_g_Faecalibaculum*, and *uncultured_bacterium_g_Prevotellaceae_UCG-001* decreased significantly. Moreover, the abundance of pathogenic bacteria or opportunistic pathogenic bacteria such as *unclassified_f_Oscillospiraceae* also decreased significantly (Fig. 4C). In addition, the abundance of *unclassified_g_norank_f_norank_o_Gastranaerophilales* and *uncultured_bacterium_g_norank_f_norank_o_Rhodospirillales* decreased continuously on 1 d and 3 d post infection. The abundance of *uncultured_organism_g_norank_f_Muribaculaceae* decreased significantly on 1 d and 7 d post infection, while the abundance of *unclassified_g_Lactobacillus* increased significantly (Fig. 4A through C). LEfSe analysis showed significant differences in microbial species at the level of genus (Fig. 4D).

### Annotation and functional enrichment in the gut DEGs of *C. sinensis* infected mice

**FIG 5:**
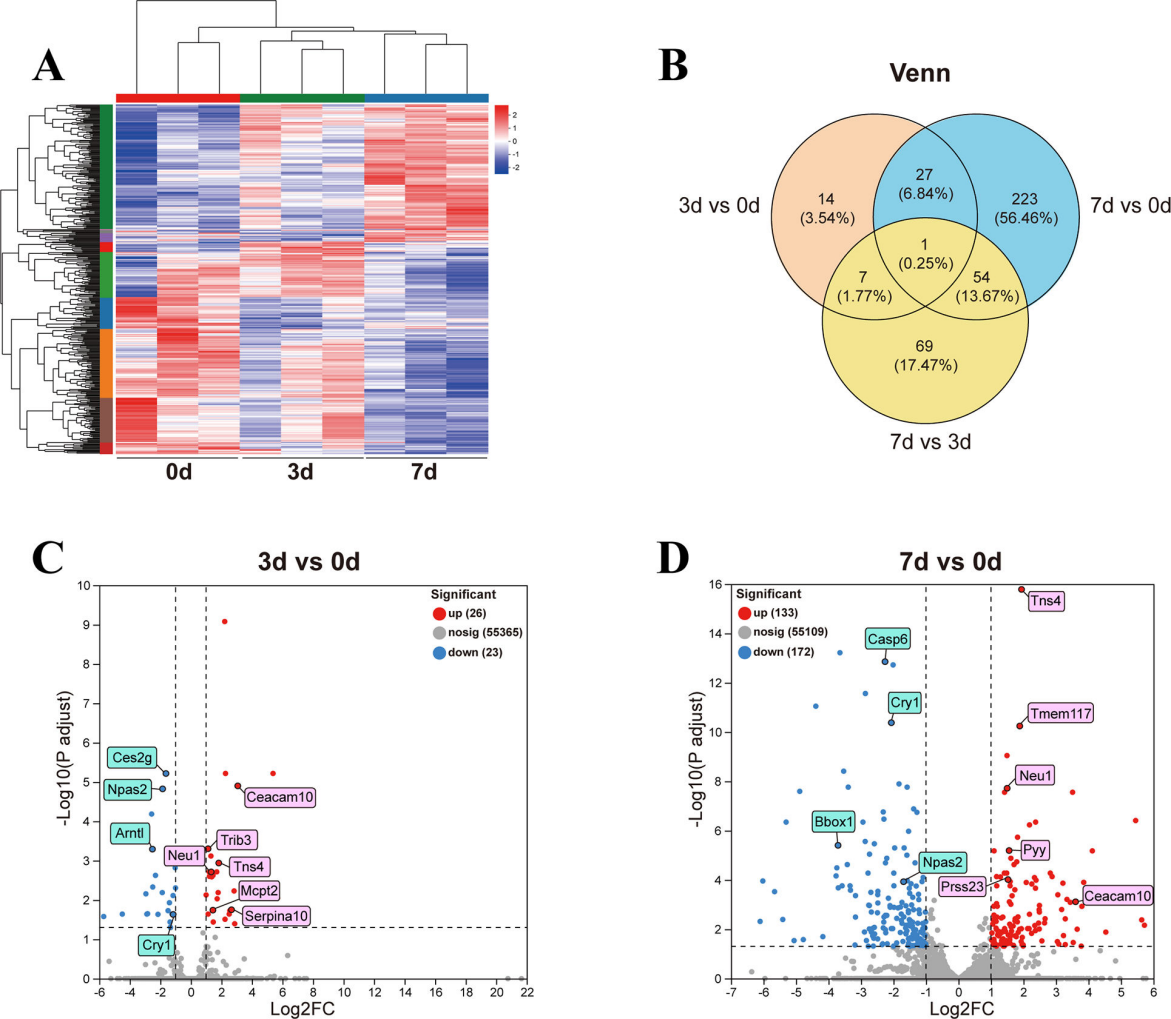
Clustering and volcano plot analysis of gut DEGs at different time points post *C. sinensis* infection. (**A**) Expression of gut genes at various time points. (**B**) Venn analysis of DEGs for 3 d vs 0 d, 7 d vs 0 d, and 7 d vs 3 d. (**C**) Volcano plot of DEGs between 3 d and 0 d. (**D**) Volcano plot of DEGs between 7 d and 0 d.

The PCA results indicated that as the infection time increased, the samples among groups gradually became more dispersed (Fig. S2A). Venn analysis presented the total number of genes detected in each group (Fig. S2B). The heatmap showed the expression levels of all detected DEGs in each group (Fig. 5A). The Venn analysis revealed the common and unique genes among different sets of target genes (Fig. 5B). A total of 49 DEGs (26 up- and 23 downregulated) and 305 DEGs (133 up- and 172 downregulated) were detected on 3 d vs 0 d and 7 d vs 0 d, respectively (|logFC| ≥ 2, *P* < 0.05). Representative upregulated/downregulated DEGs were labeled in pink rectangles and blue rectangles, respectively (Fig. 5C and D).

**FIG 6:**
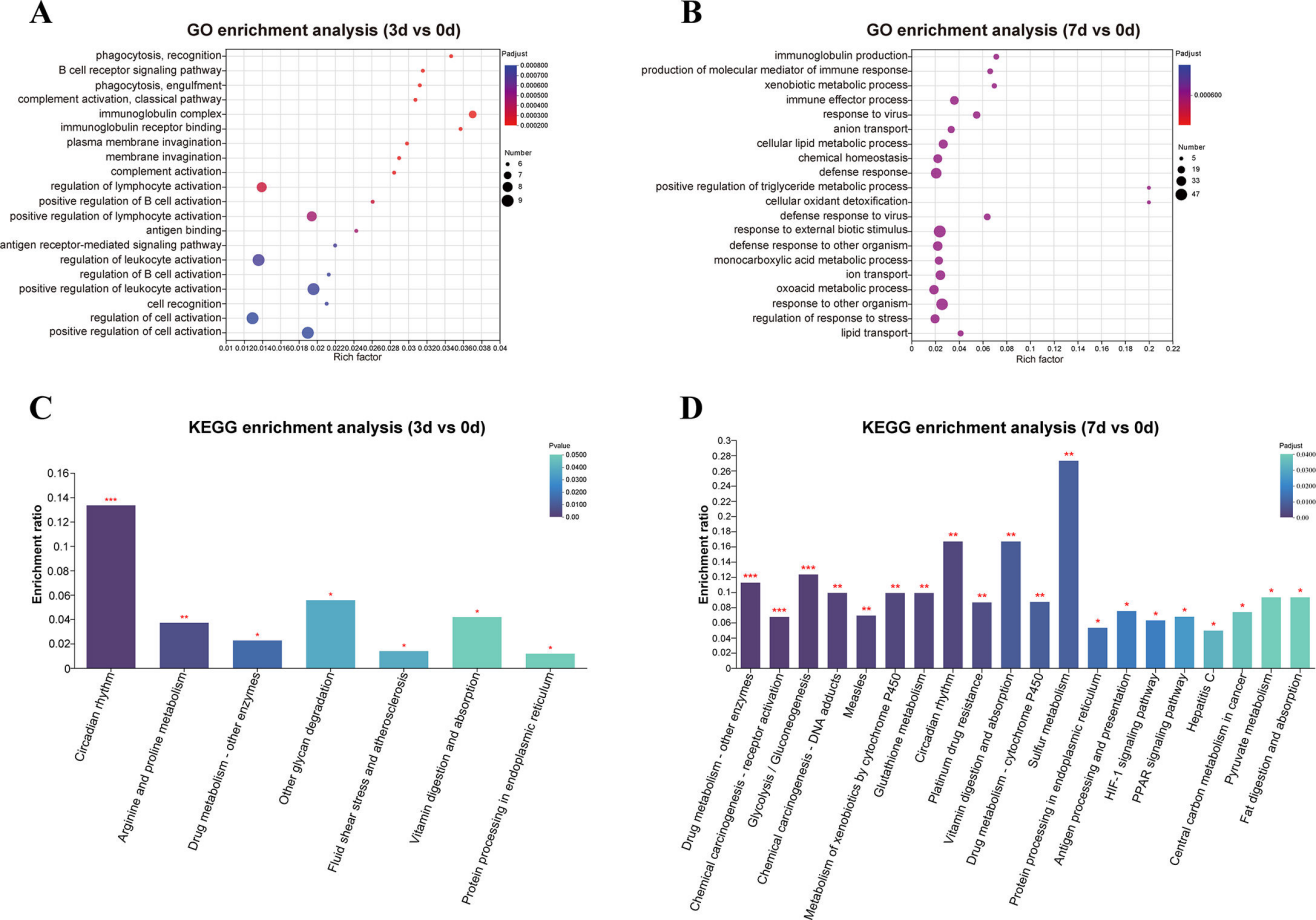
GO and KEGG enrichment analysis of DEGs in the mouse intestine at different time points post *C. sinensis* infection. (**A**) GO enrichment analysis of DEGs between 3 d and 0 d. (**B**) GO enrichment analysis of DEGs between 7 d and 0 d. (**C**) KEGG pathway enrichment analysis of DEGs between 3 d and 0 d. (**D**) KEGG pathway enrichment analysis of DEGs between 7 d and 0 d.

The GO enrichment of 3 d vs 0 d mainly involved immune aspects, such as phagocytosis, recognition, B cell receptor signaling pathway, phagocytosis, engulfment, complement activation, classical pathway, and immunoglobulin complex (Fig. 6A). The GO enrichment of 7 d vs 0 d also involved immune aspects, such as immunoglobulin production, production of molecular mediator of immune response, etc. In addition, GO terms about lipid metabolism and detoxification, such as positive regulation of triglyceride metabolic process, cellular lipid metabolic process, and cellular oxidant detoxification, were also enriched (Fig. 6B). The top three enriched KEGG pathways of 3 d vs 0 d were circadian rhythm, arginine and proline metabolism, and drug metabolism-other enzymes (Fig. 6C). The main enriched KEGG pathways of 7 d vs 0 d were drug metabolism-other enzymes, chemical carcinogenesis-receptor activation, glycolysis/gluconeogenesis, chemical carcinogenesis-DNA adducts, and circadian rhythm (Fig. 6D).

### Correlation analysis between gut microbiota and transcriptome

**FIG 7:**
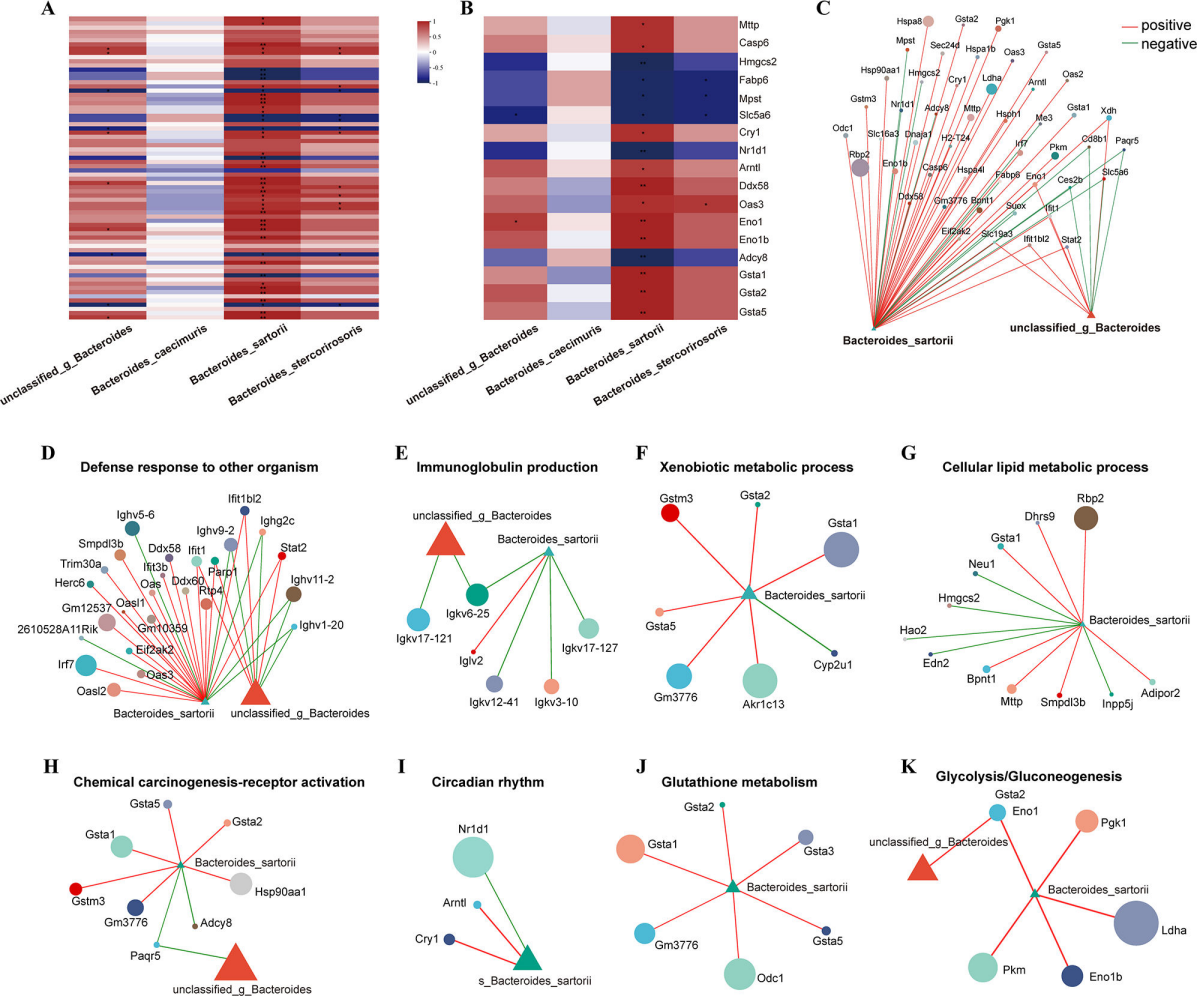
The correlation analysis between gut microbiota and transcriptome. (**A**) Correlation analysis between gut microbiota and the expression of total DEGs. (**B**) Correlation analysis between gut microbiota and important DEGs. (**C**) Correlation network between gut microbiota and total DEGs. (**D–G**) The relationship between important GO pathways and gut microbiota. (**H–K**) The relationship between important KEGG pathways and gut microbiota.

To further research the relationship and interaction between gut microbiota and transcriptome, the correlation and significance between gut microbiota and DEGs of 7 d vs 0 d were analyzed. Heatmap displayed the relationship among correlated DEGs and four bacteria, namely *unclassified_g_Bacteroides*, *Bacteroides_caecimuris*, *Bacteroides_sartorii* and *Bacteroides_stercorirosoris* (Fig. 7A), and the representative DEGs were mainly significantly related to *Bacteroides_sartorii* and *Bacteroides_stercorirosoris* (Fig. 7B and C). The detailed results are shown in Table S1. The correlation network diagram displayed that DEGs such as Mpst, Nr1d1, Cry1, and Rbp2 were significantly correlated with *Bacteroides_sartorii*. Ces2b, Cd8b1, and Paqr5 were significantly negatively correlated with both *Bacteroides_sartorii* and *unclassified_g_Bacteroides*. Additionally, Eno1 and Stat2 were significantly positively correlated with them (Fig. 7C).

Further analysis of the relationship between DEGs-enriched GO items and gut microbiota showed that *Bacteroides_sartorii* was closely positively correlated with DEGs enriched in defense response to other organisms (Fig. 7D). *Bacteroides_sartorii* and *unclassified_g_Bacteroides* showed almost a negative correlation with all DEGs enriched in immunoglobulin production (Fig. 7E). In the xenobiotic metabolic process, the detoxification-related genes of Gstm3, Gsta1, Gsta2 and Gsta5 were positively related to *Bacteroides_sartorii* (Fig. 7F). In the cellular lipid metabolism process, *Bacteroides_sartorii* was positively correlated with lipid metabolism-related gene of Mttp, cancer-related genes of Rbp2, and Dhrs9, but was also negatively correlated with cancer-related genes of Neu1 and Hmgcs2 (Fig. 7G). In addition, significantly correlated KEGG pathways, namely chemical carcinogenesis-receptor activation, circadian rhythm, glutathione metabolism, and glycolysis/gluconeogenesis, were selected (Fig. 7H through K). Among them, the DEGs involved in glutathione metabolism and glycolysis/gluconeogenesis, such as Gsta1, Gsta2, Eno1, and Eno1b, were positively correlated with *Bacteroides_sartorii* (Fig. 7J and K).

## DISCUSSION

The *C. sinensis* juvenile parasitizes the bile ducts of the definitive host, and its *Cs*ESPs can lead to hepatic inflammation, liver fibrosis, and even hepatobiliary carcinoma (21, 22). In the enterohepatic circulation, exogenous/endogenous substances can enter the intestines through the bile duct or systemic circulation. Similarly, substances produced in the intestines can enter the liver through systemic circulation or the portal vein, thereby forming enterohepatic crosstalk (23). Histological analysis of the intestine revealed that prolonged infection with *C. sinensis* juvenile resulted in elongated villi in the small intestine, as well as an increase in the number of goblet cells and acidic mucus. The elongation of gut villi is associated with improved nutrient absorption (24). These results suggested that *C. sinensis* juvenile infection can stimulate the gut immune response and barrier resistance. Moreover, these changes are consistent with the increased abundance of *Lactobacillus* observed in our study. *Lactobacillus* is a beneficial bacterium that provides nutrients to the intestines and helps maintain the integrity and stability of the gut barrier (25, 26). Moreover, the mucous layer secreted by goblet cells acts as a physical barrier separating gut microbiota from host cells. In the event of pathogen invasion, goblet cells secrete gut mucus to protect the intestines (27, 28). Therefore, early infection with *C. sinensis* juvenile stimulates the immune response in the mouse gut and enhances the resistance of the gut barrier.

The gut is considered the largest immune organ, and a healthy intestine is characterized by a well-structured barrier, efficient absorption and immune functions, and a diverse and balanced microbiota (29). Furthermore, the loss of diversity in the gut microbiota is the most common symptom of gut diseases (30). In our study, significant alterations in both alpha diversity and beta diversity indicated that *C. sinensis* juvenile infection affected the richness and diversity of the gut microbiota in mice, particularly on 7 d post-infection. In both mice and humans, the dominant phyla in the gut microbiota are Firmicutes and Bacteroidetes (31). Previous research has shown a correlation between the Firmicutes to Bacteroidetes (F/B) ratio and obesity, blood glucose levels, and body weight (32). In our study, we observed varying degrees of increase in the abundance of Firmicutes with longer infection duration, accompanied by a decrease in Bacteroidetes. Consequently, the F/B ratio increased after infection, suggesting a shift towards a diseased state.

At the genus level, we observed a decrease in the abundance of beneficial bacteria such as *unclassified_g_Akkermansia* and *unclassified_g_Bacteroides* on 1 d post-infection (33, 34). As the infection time increased, this impact became more pronounced, particularly on 7 d post-infection. Additionally, the abundance of pathogenic bacteria, such as *unclassified_f_Oscillospiraceae*, was also affected by the infection (35). Furthermore, we observed a decrease in the abundance of *norank_f_Muribaculaceae* within the Bacteroidetes phylum, particularly on 7 d after infection. *Muribaculaceae* is known to produce SCFAs and participate in oxidative stress and gut protection (36). Its decrease in abundance has been observed in various diseases, including inflammatory bowel disease (IBD) (36–38). Moreover, we found that the abundance of *Akkermansia,* a probiotic present in the mucosal layer of the mammalian intestine, also showed significant changes (39). It significantly decreased on 1 d after infection and was alleviated on 3 d and 7 d. A previous study showed that supplementation with *Akkermansia* can help eliminate parasites in mice infected with *T. gondii*, indicating its important role in protecting the intestine from parasitic invasion (33). Taken together, these results indicate that *C. sinensis* juvenile infection could significantly increase the ratio of F/B and change the composition of bacteria at the genus level in the mouse gut.

In the analysis of differential bacteria, we observed significant impacts on probiotics after 1 day of infection compared to the control group. Specifically, the abundance of *Akkermansia*, *Bacteroides*, and *Muribaculaceae*, which are known to produce SCFAs (33, 34, 36), was significantly decreased, while the abundance of pathogenic bacteria such as *Gemella* and the opportunistic pathogen *Alistipes* was significantly increased (40, 41). SCFAs are anti-inflammatory and can activate Peroxisome Proliferator-Activated Receptor Gamma (PPAR-γ) to maintain anaerobic conditions in the intestine (42–44). Conversely, the abundance of *Alistipes*, an opportunistic pathogen, was increased, which may promote chronic inflammation when the intestinal barrier is damaged (45, 46). It was worth noting that the dysbiosis of the gut microbiota was alleviated 3 d post-infection, possibly due to the adaptation of the mice to the infection of *C. sinensis* juvenile. However, it worsened after 7 d of infection, with more significant changes in differential bacteria observed. Among them, the bacteria involved in immune and anti-inflammatory responses, such as *Prevotellaceae_UCG-001*, *Muribaculum*, and *Muribaculaceae*, were decreased in abundance. *Prevotellaceae_UCG-001* is a beneficial bacterium with anti-inflammatory effects that can alleviate metabolic disorders (47). In addition, it has also been reported to be associated with gut inflammation in rodents (48). *Muribaculum* contributes to energy homeostasis by producing SCFAs and regulating carbohydrate metabolism (49–51). Therefore, infection with *C. sinensis* juvenile reduced the richness and diversity of the gut microbiota in mice, particularly lowering the abundance of beneficial bacteria, and increasing the abundance of pathogenic bacteria, leading to dysbiosis.

Our gut transcriptomic analysis revealed the enrichment of abundant immune-related GO terms, such as regulation of lymphocyte activation, positive regulation of B cell activation, and positive regulation of lymphocyte activation. The DEGs involved in these GO terms, including Igkv12-41, Ighv8-12, Ighv1-4, and Ighv9-1, were significantly upregulated. All these DEGs belong to immunoglobulins and played crucial roles in the immune function of body (52). Moreover, on 3 d and 7 d post-infection, DEGs related to gut diseases were significantly affected, particularly Ceacam10, Tns4, Neu1, Serpina10, and Npas2. Ceacam10, a member of the Ceacam family, has been reported to mediate cell proliferation (53). Meanwhile, genes related to cancer metastasis, such as Tns4, Neu1, Serpina10, and Trib3 (54–57), as well as Mcpt1 and Mcpt2, which are associated with parasitic infections, were significantly upregulated after infection. Mcpt1 is a main component of secretory granules released by mast cells during the inflammatory response, and it participates in immune reactions (58). Mcpt2 contributes to neutrophil recruitment and promotes the release of pro-inflammatory chemokines (59). KEGG analysis demonstrated that circadian rhythm pathway was significantly enriched on both 3 d and 7 d, genes related to disease/tumor, including Tns4 and Neu1 (upregulated), as well as Npas2 and Cry1 (downregulated), exhibited obvious changes. Moreover, the enrichment of metabolic pathways increased with prolonged infection, including glycolysis/gluconeogenesis, metabolism of xenobiotics by cytochrome P450, glutathione metabolism, and pyruvate metabolism. These findings indicated that *C. sinensis* invasion significantly alters the expression of intestinal genes related to immunity and diseases, disrupts the biological rhythm of intestinal tissue, and leads to metabolic disorders.

On 7 d after infection, Spearman correlation analysis was conducted to verify the significant correlation between the decreased abundance of *Bacteroides_sartorii* and detoxification, metabolism, immunity, and rhythm-related GO items and pathways, particularly the metabolic and detoxification-related pathways. A previous study reported that *Bacteroides_sartorii* has the potential to improve glucose metabolism and combat obesity (60). The DEGs enriched in glutathione metabolism and glycolysis/gluconeogenesis pathways, including Gsta1, Gsta2, and Gsta3, are crucial molecules involved in the detoxification and metabolic processes (61, 62). These DEGs were found to have a positive correlated with *Bacteroides_sartorii*, which is consistent with a previous report (60). Additionally, *Bacteroides_sartorii* also exhibited a significant positive correlation with DEGs of Cry1 and Arntl, and a significant negative correlation with Nr1d1 within the circadian rhythm pathway. Cry1, Nr1d1, and Arntl are typically involved in regulating sleep cycles and metabolic functions (63–65), and study suggests that circadian rhythms may be a key factor in maintaining a balanced gut microbiota (66). These findings indicated that after *C. sinensis* infection, *Bacteroides_sartorii* may play a crucial role in connecting gut microbiota and transcriptome interactions, primarily by influencing genes related to immunity, metabolism, detoxification, and circadian rhythms. This study indicates that *Bacteroides sartorii* may play a crucial role in the interaction between the gut microbiome and the host’s transcriptome of host following *C. sinensis* infection. However, the exact mechanisms underlying this interaction remain enigmatic, necessitating further investigation.

### Conclusions

**FIG 8:**
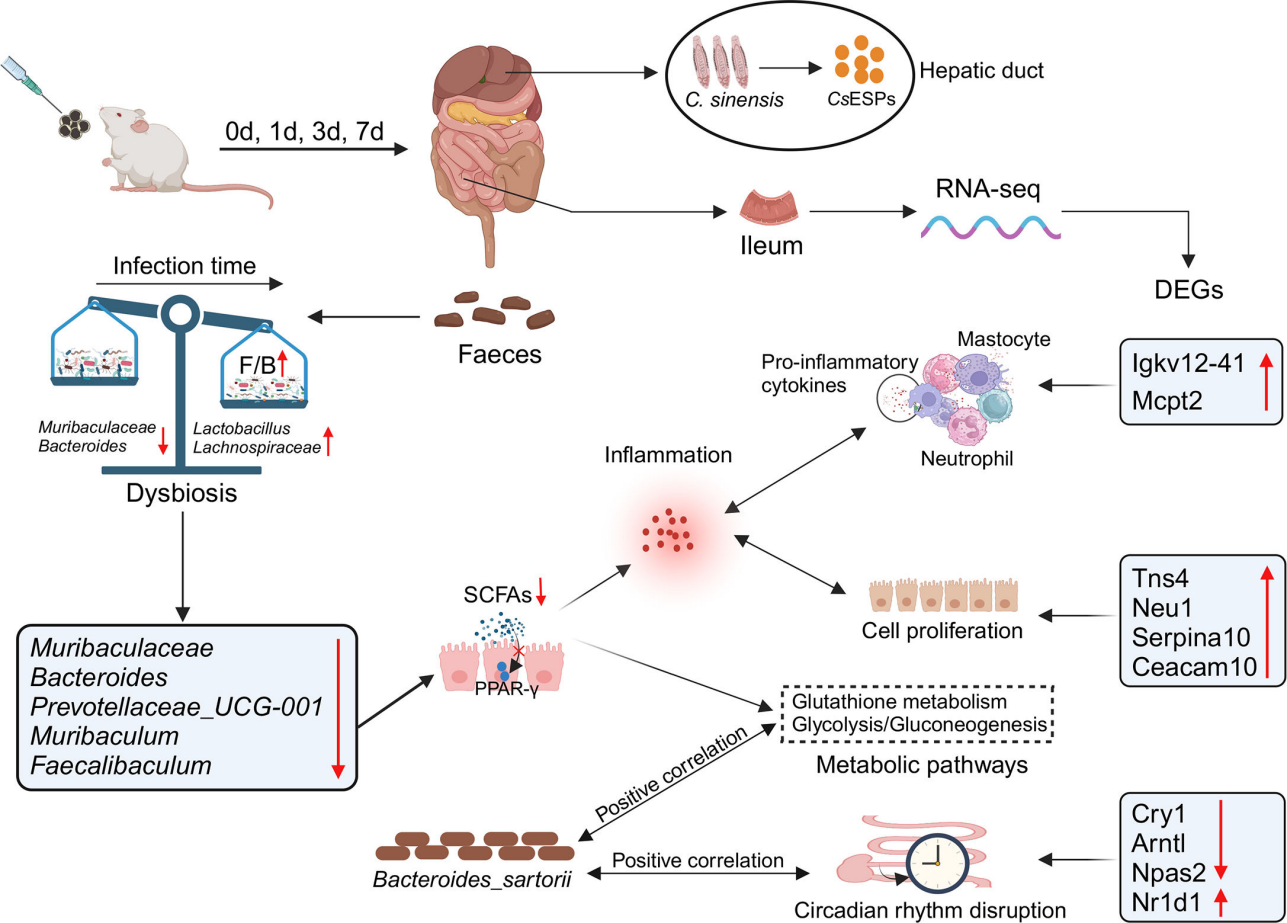
The impact of *C. sinensis* infection on the gut microbiota and transcriptome of mice. Infection with *C. sinensis* significantly affected the abundance of beneficial bacteria in the gut, upregulated genes related to immunity, disease, and circadian rhythm, thereby affecting the host’s immune response and metabolic processes. Meanwhile, the interaction between *Bacteroides* and the gut transcriptome may play a crucial role in *C. sinensis* infection. Created with BioRender.com.

In summary, infection with *C. sinensis* juvenile could stimulate the growth of intestinal villi and increase the number of goblet cells in mice. It also affected the abundance of gut microbiota that produce SCFAs (e.g., *Lactobacillus*, *Muribaculaceae*, *Bacteroides,* and *Prevotellaceae_UCG-001*), as well as gut transcription levels. With increasing infection time, abundant immune and detoxification-related GO items were enriched, such as immunoglobulin production, xenobiotic metabolic process, and defense response to other organisms. In addition to the co-enriched circadian rhythm, more detoxification and metabolism KEGG pathways were enriched, such as glutathione metabolism and glycolysis/gluconeogenesis. Furthermore, the interaction between *Bacteroides_sartorii* and genes related to immunity, metabolism, and circadian rhythm plays a crucial role in *C. sinensis* juvenile infection (Fig. 8). These findings contribute to further research on the mutual influence mechanism between intestinal microbiota and transcriptome of *C. sinensis* juvenile invasion.

## Data Availability

The raw RNA-seq data has been deposited to NCBI database under the accession number PRJNA1105322 and PRJNA1111484.

## SUPPLEMENTAL MATERIAL

Figures S1 and S2 spectrum.01550-24-s0001.docx Fig. S1: Gut microbial richness of mice infected with *C. sinensis* at different time points. Fig. S2: Effect of *C. sinensis* infection on the mouse gut transcriptome.

Tables S1 spectrum.01550-24-s0002.xlsx The correlation *P* value between gut microbiota and DEGs (7 d vs 0 d).

ASM does not own the copyrights to Supplemental Material that may be linked to, or accessed through, an article. The authors have granted ASM a non-exclusive, world-wide license to publish the Supplemental Material files. Please contact the corresponding author directly for reuse.
