# Supplementary material for: Correlation analysis of the impact of Clonorchis sinensis juvenile on gut microbiota and transcriptome in mice
Source: Microbiol Spectr. 2024 Dec 27;13(2):e01550-24. doi: 10.1128/spectrum.01550-24 (PMC11792474; doi:10.1128/spectrum.01550-24)
Supplement: Figures S1 and S2 — Fig. S1: Gut microbial richness of mice infected with C. sinensis at different time points. Fig. S2: Effect of C. sinensis infection on the mouse gut transcriptome. [file spectrum.01550-24-s0001.docx]

# Supplementary information


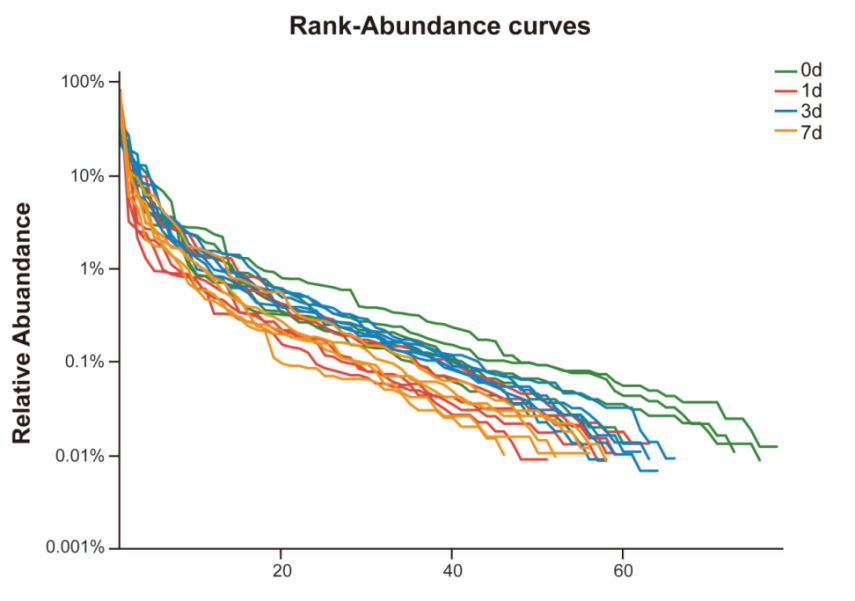


**Fig.** **S1** Gut microbial richness of mice infected with *C. sinensis* at different time points.


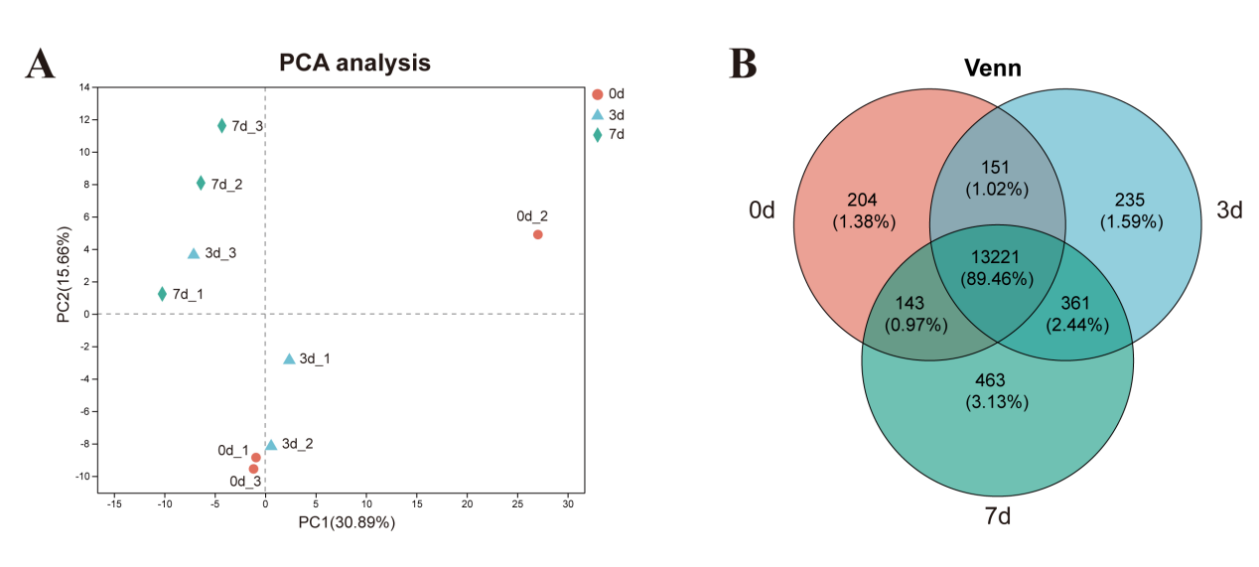


**Fig. S2** Effect of *C. sinensis* infection on the mouse gut transcriptome. (A) PCA analysis of 0 d, 3 d, and 7 d groups. (B) Venn analysis of genes identified in the 0 d, 3 d, and 7 d groups.
